# No genetic causal association between systemic lupus erythematosus and COVID-19

**DOI:** 10.3389/fimmu.2023.1183570

**Published:** 2023-05-18

**Authors:** Shu-Zhen Xu, Zhi-Xin Wang, Xi Fang, Cong Chen, Xiao-Ke Yang, Zong-Wen Shuai, Sha-Sha Tao

**Affiliations:** ^1^ Department of Epidemiology and Biostatistics, School of Public Health, Anhui Medical University, Hefei, Anhui, China; ^2^ Institute of Kidney Disease, Inflammation and Immunity Mediated Diseases, The Second Hospital of Anhui Medical University, Hefei, Anhui, China; ^3^ Department of Rheumatology and Immunology, The First Affiliated Hospital of Anhui Medical University, Hefei, Anhui, China; ^4^ Experimental Teaching Center for Preventive Medicine, School of Public Health, Anhui Medical University, Hefei, Anhui, China

**Keywords:** systemic lupus erythematosus, COVID-19, genetic association, causal relationship, Mendelian randomization

## Abstract

**Objective:**

Emerging evidence suggests an increased prevalence of coronavirus disease 2019 (COVID-19) in patients with systemic lupus erythematosus (SLE), the prototype of autoimmune disease, compared to the general population. However, the conclusions were inconsistent, and the causal relationship between COVID-19 and SLE remains unknown.

**Methods:**

In this study, we aimed to evaluate the bidirectional causal relationship between COVID-19 and SLE using bidirectional Mendelian randomization (MR) analysis, including MR-Egger, weighted median, weighted mode, and the inverse variance weighting (IVW) method.

**Results:**

The results of IVW showed a negative effect of SLE on severe COVID-19 (OR = 0.962, *p* = 0.040) and COVID-19 infection (OR = 0.988, *p* = 0.025), which disappeared after Bonferroni correction. No causal effect of SLE on hospitalized COVID-19 was observed (OR = 0.983, *p* = 0.148). In the reverse analysis, no causal effects of severe COVID-19 infection (OR = 1.045, *p* = 0.664), hospitalized COVID-19 (OR = 0.872, *p* = 0.109), and COVID-19 infection (OR = 0.943, *p* = 0.811) on SLE were found.

**Conclusion:**

The findings of our bidirectional causal inference analysis did not support a genetically predicted causal relationship between SLE and COVID-19; thus, their association observed in previous observational studies may have been caused by confounding factors.

## 1 Introduction

In the past 3 years, coronavirus disease 2019 (COVID-19), caused by severe acute respiratory syndrome coronavirus 2 (SARS-CoV-2), has spread all over the world and has caused high concern worldwide (1). Currently, the COVID-19 pandemic is still evolving, with a high societal burden of morbidity and mortality. At the time of writing, more than 500 million cases and approximately 6 million deaths have been reported (https://covid19.who.int/). The clinical manifestations of patients with COVID-19 are diverse, ranging from severe cases to mild and asymptomatic cases (2). Moreover, patients with COVID-19 are often observed to experience multiple complications, including interstitial pneumonia, cytopenia, arthralgia, myocarditis, and autoimmune diseases (3–5).

In addition to complications during illness, late-onset complications in patients with COVID-19 are also increasingly reported, among which autoimmune manifestations have attracted much attention (4–13). Emerging reports have suggested that COVID-19 may lead to autoimmune and autoinflammatory diseases, in turn leading patients with COVID-19 to enter a vicious circle of infection and to be closely associated with increased morbidity and mortality (4, 11, 14). Among these studies, great attention has been focused on systemic lupus erythematosus (SLE), the prototypical autoimmune disease (7–9).

SLE is a chronic systemic autoimmune disorder whose pathogenesis is complex and characterized by the production of multiple autoantibodies and immune complex deposition (15). Globally, the prevalence of SLE in adults is estimated at 30–150 per 100,000, and its incidence ranges from 2.2 to 23.1 per 100,000 per year (16). Despite advances in treatment, SLE is still a cause of premature death, with a global standardized mortality rate of 2.0–5.9 (17, 18). SLE is a lifelong chronic disease that cannot be cured, yet. The accumulation of organ damage and the side effects of the long-term use of hormones and immunosuppressive drugs seriously affect the quality of life of patients and bring significant economic burdens to them (19, 20).

Recently, the association between COVID-19 and SLE has attracted a lot of attention. Some studies found that the prevalence of COVID-19 is increased in patients with SLE compared to the general population (21, 22). However, conclusions about the prevalence of COVID-19 in patients with SLE have been inconsistent. There are also studies indicating that the prevalence of COVID-19 is similar between SLE patients and normal controls (12, 13), while some studies even reported a decrease in COVID-19 prevalence in patients with SLE compared with the general population (23, 24). These causal inferences from observational studies are limited and unreliable, which could be due to the influence of unmeasured or unknown confounding factors (25). On the other hand, due to the serious adverse effects of COVID-19, randomized controlled trials (RCTs), the most commonly used tool for assessing causality, cannot be conducted to study the causal relationship between COVID-19 and these adverse health outcomes. Therefore, the causal relationship between COVID-19 and SLE remains obscure.

Clarifying the relationship between COVID-19 infection and SLE is necessary for further research on the diagnosis, treatment, and recovery of patients with SLE infected with COVID-19. Further research is also helpful for further understanding the feasibility and the risk of vaccination for patients with SLE and is also essential for the development of strategies for the treatment and care of SLE patients during a COVID-19 infection. Therefore, there is an urgent need to evaluate the causal relationship between COVID-19 and SLE (26).

Mendelian randomization (MR), an advanced study design using genetic variants as instrumental variables (IVs), has been widely used to evaluate causal relationships between exposure factors and outcomes (27). It can reduce the confounding effects from environmental factors as alleles are randomly allocated at conception. In addition, it could also avoid reverse causal bias because the genotype would not be affected by diseases (28). Furthermore, compared to RCT, MR can be conducted using existing open-access data from large-scale genome-wide association studies (GWAS), which extremely increases its scope and statistical power (29).

In this study, we conducted a bidirectional two-sample MR analysis to assess the causal relationship between COVID-19 and SLE, which is beneficial for the development of strategies for the treatment and care of patients with SLE during infection with COVID-19.

## 2 Materials and methods

### 2.1 Study design

Our summary data were obtained from published studies, all data of which had been approved by institutional review committees. A bidirectional two-sample MR method was employed to verify the causative effects between SLE and three types of COVID-19 infection.

### 2.2 Data sources and single nucleotide polymorphism selection

#### 2.2.1 GWAS of COVID-19

The COVID-19-related data were obtained from the COVID-19 host genetics initiative GWAS (release 5) (https://www.covid19hg.org/results/) (30). We selected data from studies in which all participants were from the European population and the whole population was used as the control. We assessed the causality between three different populations with COVID-19 infection and SLE: COVID-19 infection (total cases = 38,984, total controls = 1,644,784), hospitalized COVID-19 (total cases = 9,986, total controls = 1,877,672), and severe COVID-19 (total cases = 5,101, total controls = 1,383,241).

#### 2.2.2 GWAS of SLE

Genetic associations of SLE were retrieved from the largest public GWAS meta-analysis of Bentham et al. (31), which included 7,219 cases and 15,991 controls. The corresponding genetic information of the single nucleotide polymorphisms (SNPs) of the three different COVID-9 infection groups was reviewed and collected in an SLE consortium.

#### 2.2.3 SNP selection

First, we screened out the SNPs that had strong associations with exposure (*p* < 5 × 10^−8^) from the exposure GWAS. Second, to exclude the effect of linkage disequilibrium (LD) on the MR results, we used the clumping process (*r* ^2^ < 0.001, clumping distance = 10,000 kb) to ensure that there was no LD between SNPs. Third, SNPs with a minor allele frequency (MAF) <0.01 were excluded. Fourth, the selected SNPs were matched to the outcome GWAS, with the missing SNPs replaced by their proxy SNPs with high LD (*r* ^2^ > 0.8). Finally, after removing palindromic SNPs, the other SNPs selected were used as IVs.

### 2.3 Statistical analyses

The causal relationship between COVID-19 infection and SLE was analyzed using four highly efficient and complementary methods, namely, MR-Egger, weighted median, weighted mode, and inverse variance weighting (IVW), with IVW as the main analytical method. The IVs were assessed for potential horizontal pleiotropy using MR-Egger regression and the MR pleiotropy residual sum and outlier (MR-PRESSO) method (32, 33). In addition, MR-PRESSO can also find outliers in the IVs. After removing outliers, the MR-Egger and MR-PRESSO tests were performed again until there was no SNP with horizontal pleiotropy in all the IVs. Heterogeneity among IVs was detected and quantified using Cochran’s *Q* statistic (34). The leave-one-out sensitivity analysis was used to determine and exclude SNPs that had a strong impact on the results to ensure the reliability and stability of the causal effect estimates. Three types of COVID-19 infection were analyzed in this study, and a bidirectional two-sample MR study was conducted to assess the causal relationship between COVID-19 and SLE. The Bonferroni method was utilized to correct for multiple comparisons, and the *p*-value was <0.008 (0.05 was divided by 2*3). All analyses were carried out using the packages “TwoSampleMR” and “MRPRESSO” in R version 4.1.1.

## 3 Results

### 3.1 Instrumental variable selection

#### 3.1.1 SLE IVs

Initially, 15,984 SNPs with strong associations (*p* < 5 × 10^−8^) with SLE were screened out from the SLE GWAS data. After the clumping process, 43 SNPs with no LD were selected. None of their MAFs were less than 0.01. Data on the main information of the SNPs, including the effect allele, other alleles, beta, standard error of beta (SE), and *p*-value, were collected, as shown in **Supplementary Table S1**. rs143123127, rs9274357, and rs150180633 were not found in all three COVID-19 GWAS data. rs150180633 was replaced by its proxy SNP rs76610133, but no proxy SNPs for rs143123127 and rs9274357 could be found. Ultimately, 41 IVs were included in the MR analysis.

#### 3.1.2 Severe COVID-19 IVs

Initially, 649 SNPs with strong associations (*p* < 5 × 10^−8^) with severe COVID-19 were screened out from the severe COVID-19 GWAS data. After the clumping process, nine SNPs with no LD were selected. The MAFs of all the abovementioned SNPs were greater than 0.01. The main information on these SNPs is shown in **Supplementary Table S2**. rs35081325, rs111837807, and rs2237698 were not found in the SLE GWAS data. Therefore, rs35081325 and rs111837807 were replaced by their proxy SNPs, rs34288077 and rs143334143, respectively; however, no proxy SNP for rs2237698 could be found. rs13050728 was removed from the MR analysis due to its beta in the SLE GWAS data being 0. Ultimately, seven IVs were included in the MR analysis.

#### 3.1.3 Hospitalized COVID-19 IVs

A total of 709 SNPs with strong associations (*p* < 5 × 10^−8^) with hospitalized COVID-19 were screened out from the hospitalized COVID-19 GWAS data. After the clumping process, six SNPs with no LD were selected. The MAFs of all the abovementioned SNPs were greater than 0.01. The main information on these SNPs is shown in **Supplementary Table S2**. rs35081325 was not found in the SLE GWAS data and was therefore replaced by the proxy SNP rs34288077. rs13050728 was removed from the MR analysis due to its beta in the SLE GWAS data being 0. Ultimately, five IVs were included in the MR analysis.

#### 3.1.4 COVID-19 IVs

A total of 495 SNPs with strong associations (*p* < 5 × 10^−8^) with COVID-19 were screened out from the COVID-19 data. After the clumping process, seven SNPs with no LD were selected. The MAFs of all the abovementioned SNPs were greater than 0.01. The main information on these SNPs is shown in **Supplementary Table S2**. As palindromic SNPs were identified in the MR analysis, rs12482060 and rs757405 were removed. Ultimately, five IVs were included in the MR analysis.

### 3.2 Causal relationship between SLE and COVID-19

#### 3.2.1 SLE on COVID-19

**Figure 1:**
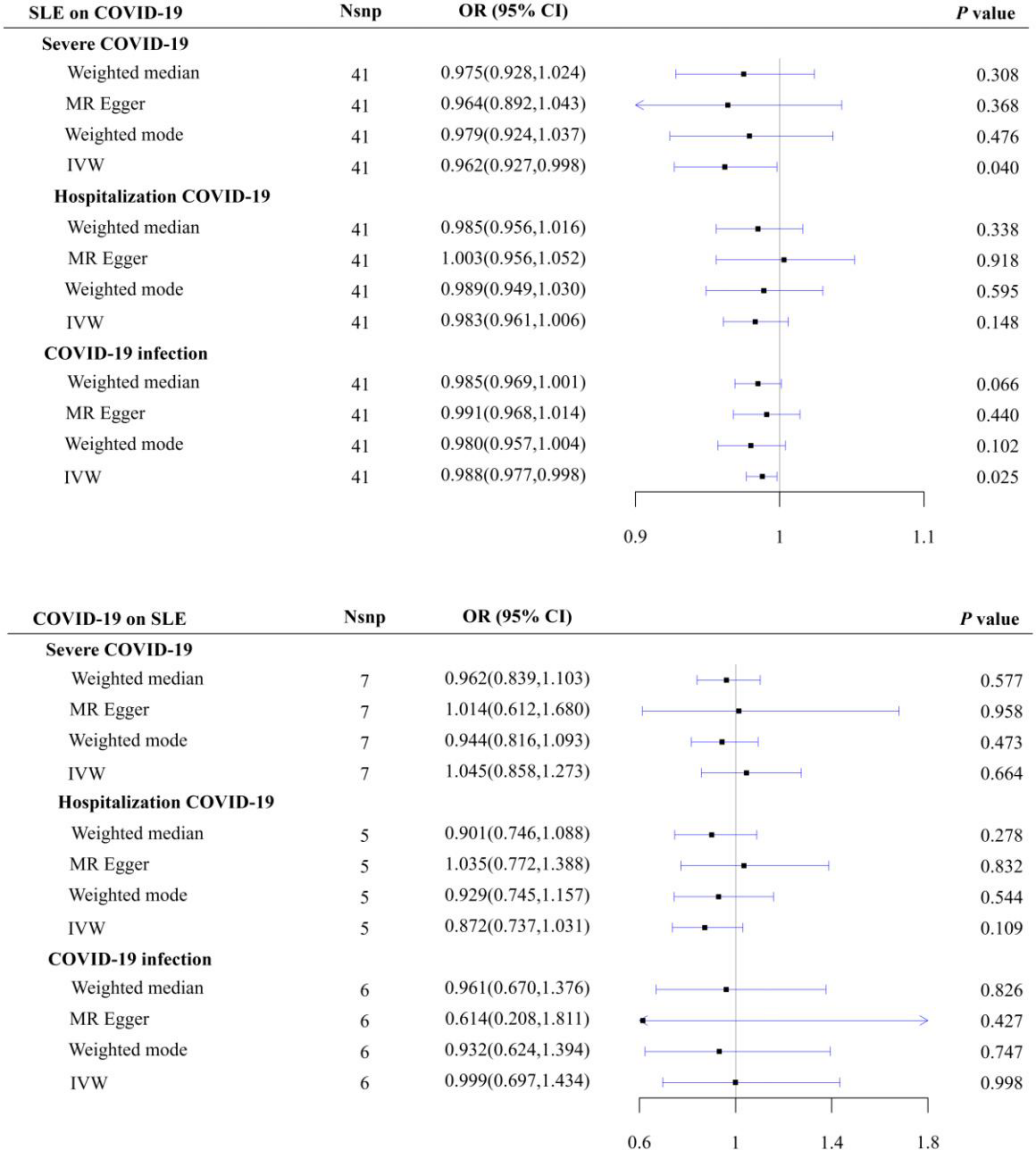
Casual relationships between SLE and COVID-19.

The results of IVW showed a negative effect of SLE on severe COVID-19 (OR = 0.962, 95% CI = 0.927–0.998, *p* = 0.040) and COVID-19 infection (OR = 0.988, 95% CI = 0.977–0.998, *p* = 0.025), which disappeared after Bonferroni correction. No causal effect of SLE on hospitalized COVID-19 was observed (OR = 0.983, 95% CI = 0.961–1.006, *p* = 0.148). The results of the MR analysis of the causal effects of SLE on COVID-19 are presented in **Figure 1** and **Supplementary Table S3**.

#### 3.2.2 COVID-19 on SLE

No causal effects of severe COVID-19 infection (OR = 1.045, 95% CI = 0.858–1.273, *p* = 0.664), hospitalized COVID-19 (OR = 0.872, 95% CI = 0.737–1.031, *p* = 0.109), and COVID-19 infection (OR = 0.999, 95% CI = 0.697–1.434, *p* = 0.998) on SLE were found. The results of the MR analysis of the causal effects of COVID-19 on SLE are shown in **Figure 1** and **Supplementary Table S4**.

### 3.3 Pleiotropy and sensitivity analysis

The heterogeneity test did not find any heterogeneity among the selected IVs. MR-Egger regression and the MR-PRESSO global test showed no horizontal pleiotropy between the IVs and outcomes, except for rs10936744, which was identified as an outlier and showed horizontal pleiotropy with SLE. After removing the outlier, the results did not change significantly (OR = 0.874, 95% CI = 0.610–1.253, *p* = 0.465) (**Supplementary Table S4**). The leave-one-out analysis suggested that the results were not driven by any SNPs. The results of the pleiotropic and sensitivity analyses are presented in **Supplementary Figures S1–S4**.

## 4 Discussion

In this study, we investigated the bidirectional causal association between SLE and COVID-19 using multiple complementary MR methods. Our two-sample MR analysis did not observe any evidence supporting the causal association of COVID-19 with SLE in individuals of European descent. Similarly, the reverse MR analysis found no evidence that genetic liability to SLE was causally related to COVID-19.

Recently, the prevalence of COVID-19 in patients with SLE has attracted a lot of attention. Some studies found that the prevalence of COVID-19 is higher in patients with SLE than in control populations (21, 22). In a study including 417 patients with SLE, 14 patients were diagnosed with COVID-19 (21). A total of 4,059 SLE patients were described in 20 research studies published before June 30, 2020, of whom 255 were diagnosed by PCR or presumed to have COVID-19 based on symptoms or radiological findings (22). However, conclusions about the prevalence of COVID in patients with SLE were inconsistent. There were studies indicating that the prevalence of COVID-19 is similar in patients with SLE compared with the general population (12, 13). In two cohort studies that included 458 and 916 SLE patients, only one and two were diagnosed with COVID-19, with SARS-CoV-2 infection rates of 0.22% and 0.21%, respectively, which were similar to that of the control population (12, 13). Moreover, there were also studies suggesting that the prevalence of COVID-19 is decreased in patients with SLE compared to the general population (23, 24). A study of 900 SLE patients conducted from 25 February to 10 April 2020 did not observe any patients infected with COVID-19 (23). In addition, only three COVID-19 cases were identified in the Asia Pacific Lupus Collaboration (APLC) patient cohort comprising 3,375 patients from 25 centers (24).

The conflicting evidence on the association between COVID-19 and SLE found in previous observational studies may be attributable to the following factors. It was demonstrated that, after functional impairment of the lung, heart, brain, and kidney tissues in the first stage, SARS-CoV-2 causes immune dysregulation and autoimmune imbalances in the second stage, as well as hormonal imbalances, which can lead to physical and mental fatigue, multi-location pains, and even autoimmune diseases, including SLE (10, 35). It was also proposed that SLE and COVID-19 shared many aspects, including some demographics of the patient populations affected and aberrant immune responses (36). On the one hand, patients with SLE may likely have a higher risk and more severe outcomes of COVID-19 not only because of their associated immunocompromised condition but also due to the immunosuppressive and cytotoxic drug treatments they received (36, 37). The abnormal innate and adaptive immunity in patients with SLE may prolong viral shedding, making them more susceptible to COVID-19 and to having a more severe infection (38, 39). DNA methylation defects can be exacerbated in SLE patients after being infected with SARS-CoV-2, which may lead to the hypomethylation of *ACE2* and the demethylation of key cytokine genes, thereby exacerbating virus-induced cytokine storms, triggering viremia and other more severe consequences (38). CD8 T-cell-mediated cytotoxicity was observed to be decreased in SLE patients, which may increase the susceptibility to and the severity of COVID-19 in these patients (39). On the other hand, patients with SLE may likely have a higher risk and severe outcomes of COVID-19 due not only to their accompanying immunocompromised state but also to the immunosuppressive and cytotoxic drug treatment received. On the other hand, there were also studies suggesting that some of the shared pathways and the use of certain steroid drugs are protective against COVID-19 in patients with SLE (40, 41). The highly active type I interferon in SLE patients was found to exert protective effects on SARS-CoV-2 infection (40). A study showed that the use of low-dose steroids can reduce the mortality of patients with severe COVID-19, suggesting that steroids may have different effects on COVID-19 depending on the dose and the disease severity (41). Another study also revealed that the risk and the outcome of COVID-19 in patients with SLE were difficult to determine due to methodological limitations, and treated with some immunosuppression did not seem to increase the susceptibility to and the severity of COVID-19 in these patients (42).

This study had several advantages. First, as a bidirectional MR study, the study assessed the possible causal relationship between COVID-19 and SLE in both directions. Second, it also evaluated the causal relationship between SLE and three different types of COVID-19 infection, which provided further insights into the causal associations between SLE and the different outcomes and severity of COVID-19 infection.

However, this study also had some limitations. First, all of the GWAS data in this study were from European populations; therefore, the representativeness of the results to the entire population remains to be determined. Second, there may be participants included in both exposure and outcome, but it was difficult to estimate the proportions of these participants. Finally, although we selected the largest GWAS database to date, the IVs of COVID-19 after screening were still somewhat underrepresented. Larger-scale COVID-19 GWAS data need to be updated.

## 5 Conclusion

The evidence from our bidirectional causal inference analysis did not support a genetically predicted causal relationship between SLE and COVID-19; thus, their association observed in previous observational studies may have been caused by confounding factors. More advanced MR analysis methods, larger-scale GWAS summary data, and more genetic instruments are needed to validate the findings of this study.

## Data availability statement

The original contributions presented in the study are included in the article/**Supplementary Material**. Further inquiries can be directed to the corresponding authors.

## Author contributions

S-ST and Z-WS conceived the present idea and were responsible for the design of the study. Z-XW participated in the acquisition of data and the data analysis. S-ZX performed the statistical analysis and wrote the manuscript. XF and CC made the figures and tables. X-KY participated in the modification of the English content. All authors contributed to the article and approved the submitted version.
