# Supplementary material for: No genetic causal association between systemic lupus erythematosus and COVID-19
Source: Front Immunol. 2023 May 18;14:1183570. doi: 10.3389/fimmu.2023.1183570 (PMC10232808; doi:10.3389/fimmu.2023.1183570)
Supplement: Supplementary file 1 [file Image_1.pdf]

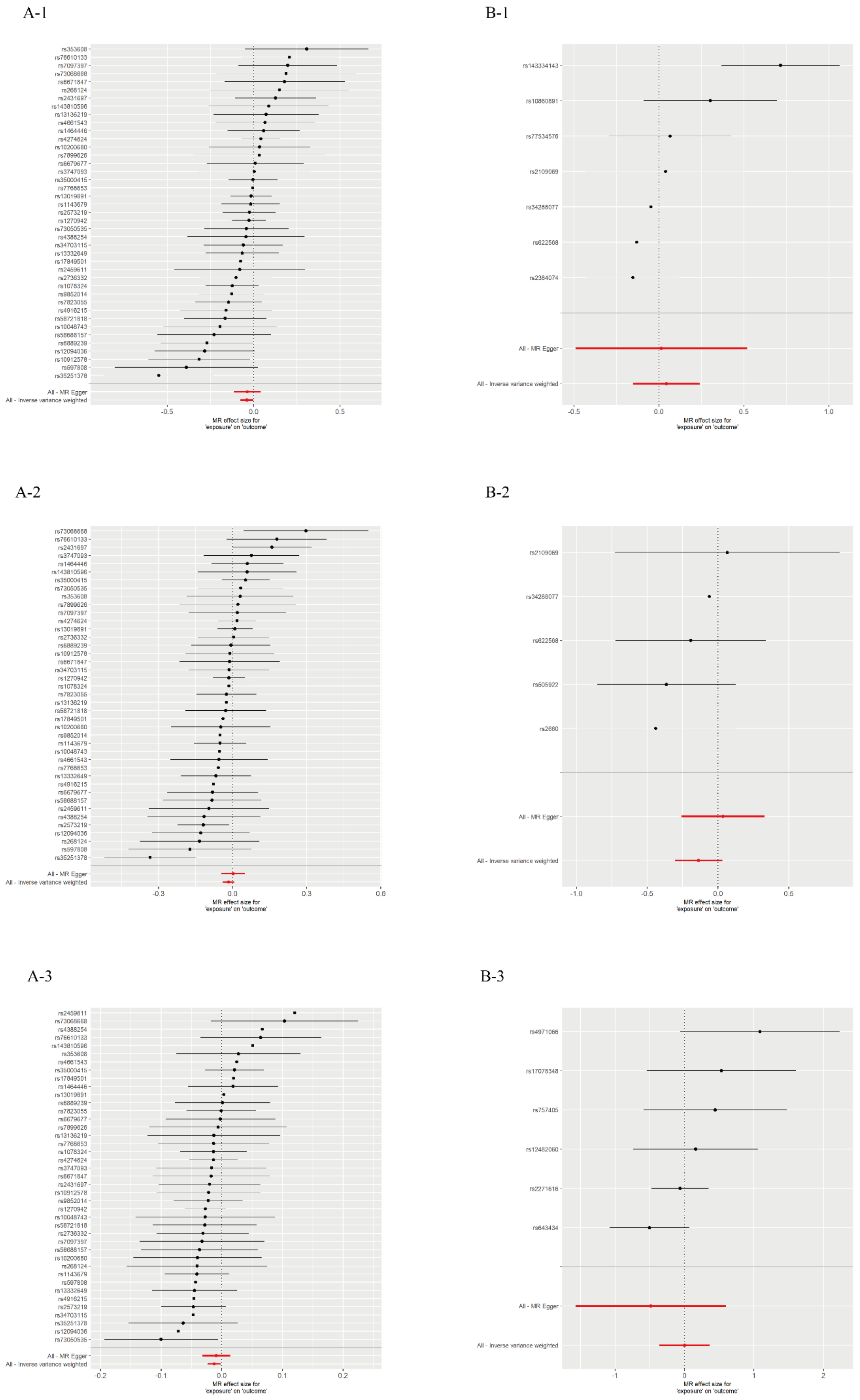

Figure S1. Forrest plot of the causal relationships between SLE and COVID-19. (A-1) MR estimates for SLE on severe COVID-19.

Legend: (A-1) MR estimates for SLE on hospitalized COVID-19. (A-3) SLE on COVID-19. (B-1) MR estimates for severe COVID-19 on SLE. (B-2) Hospitalized COVID-19 on SLE. (B-3) COVID-19 on SLE.

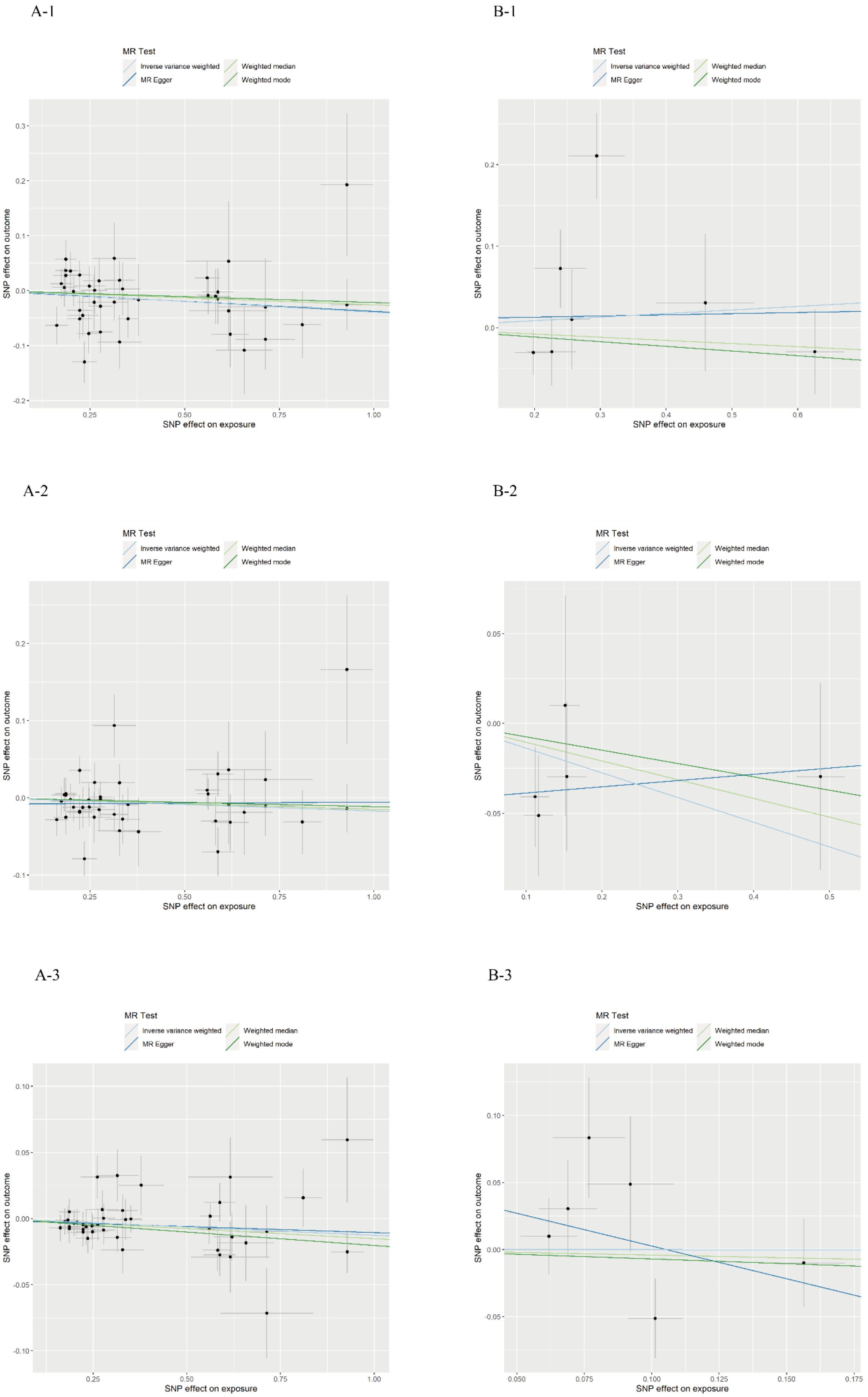

Figure S2. Scatter plot of the causal relationships between SLE and COVID-19.

Legend: (A-1) MR estimates for SLE on severe COVID-19. (A-1) MR estimates for SLE on hospitalized COVID-19. (A-3) SLE on COVID-19. (B-1) MR estimates for severe COVID-19 on SLE. (B-2) Hospitalized COVID-19 on SLE. (B-3) COVID-19 on SLE.

A-1

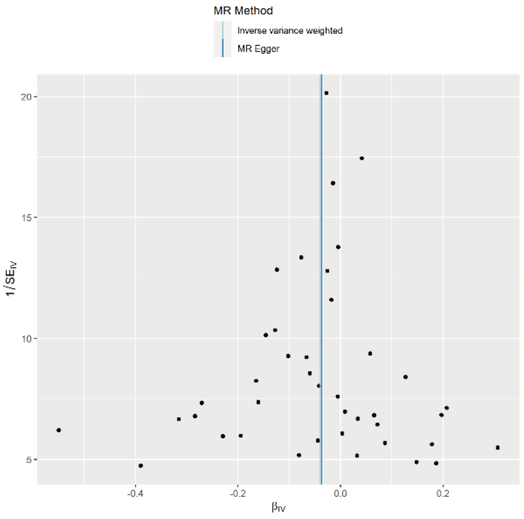

B-1

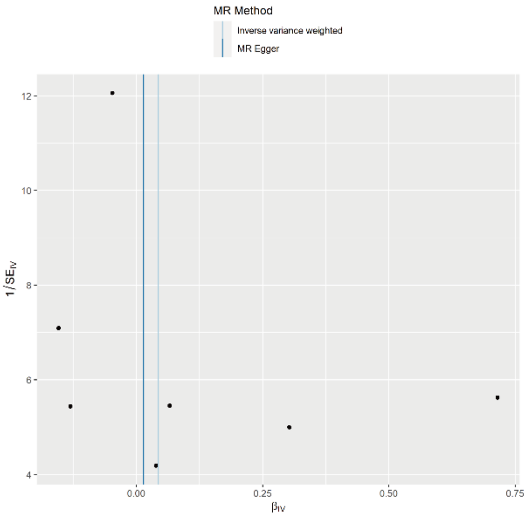

A-2

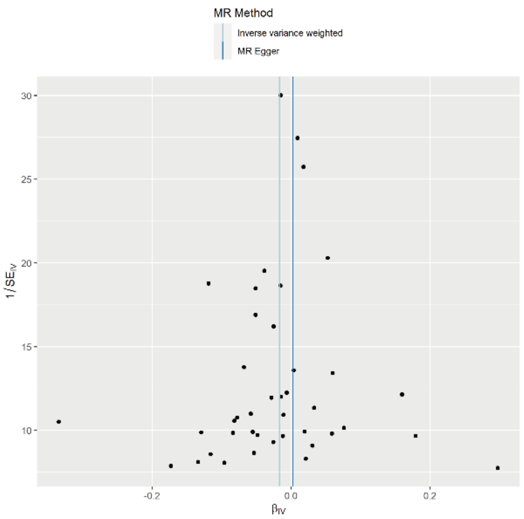

B-2

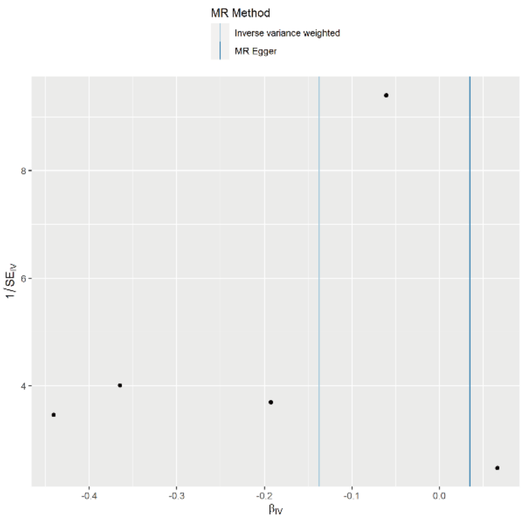

A-3

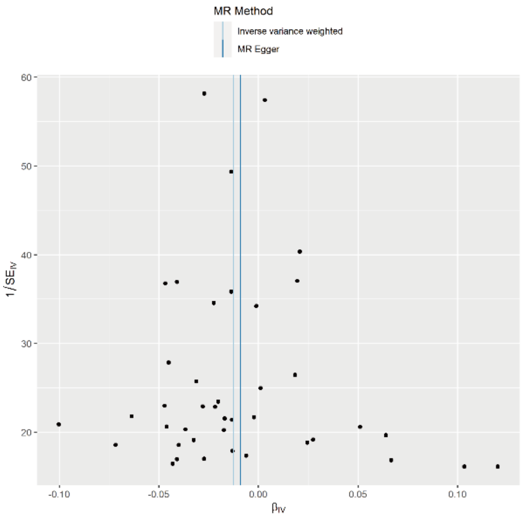

B-3

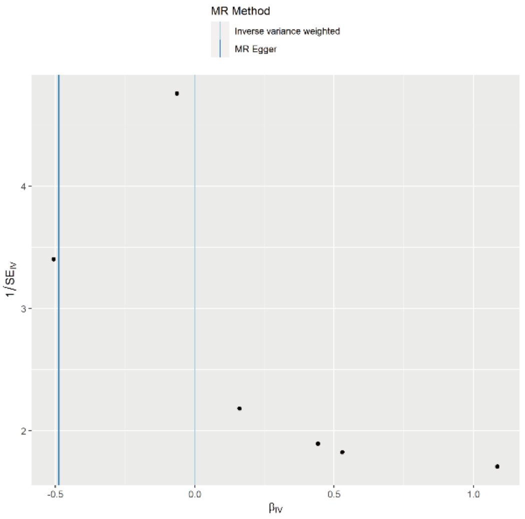

Figure S3. Funnel plot of the causal relationships between SLE and COVID-19.

Legend: (A-1) MR estimates for SLE on severe COVID-19. (A-1) MR estimates for SLE on hospitalized COVID-19. (A-3) SLE on COVID-19. (B-1) MR estimates for severe COVID-19 on SLE. (B-2) Hospitalized COVID-19 on SLE. (B-3) COVID-19 on SLE.

A-1

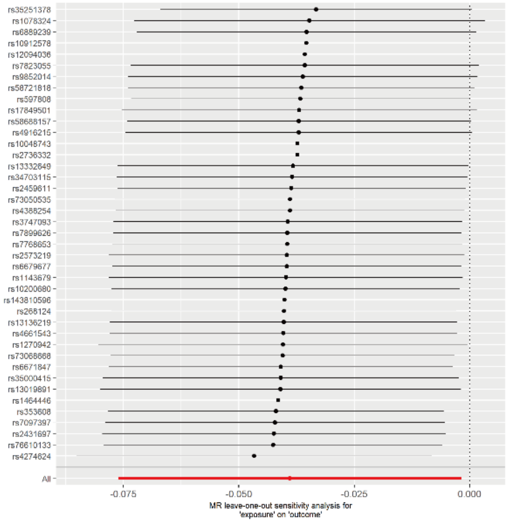

B-1

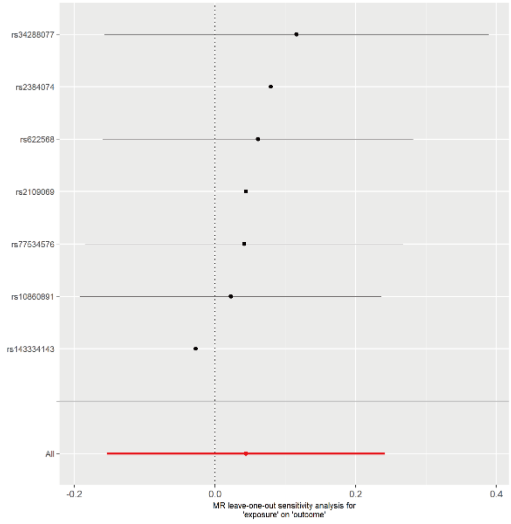

A-2

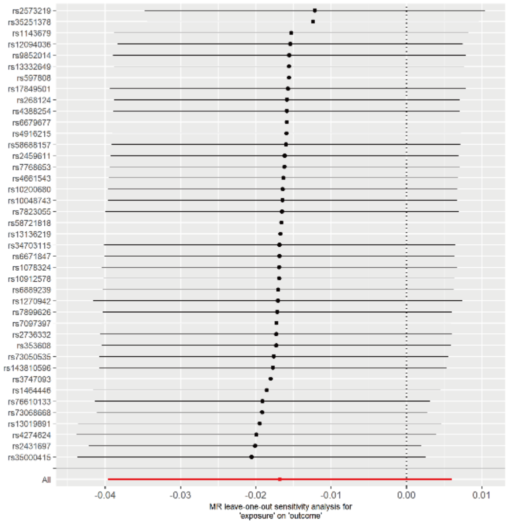

B-2

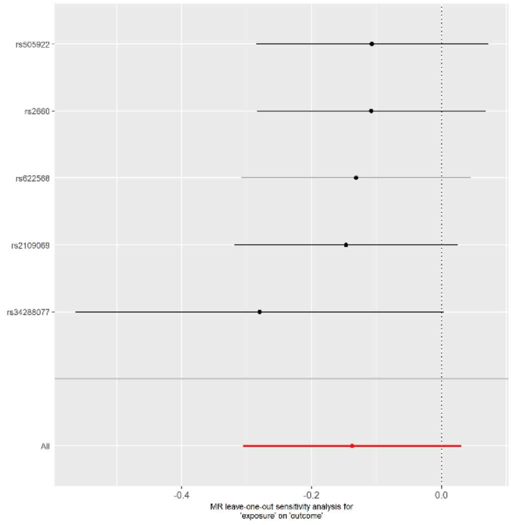

A-3

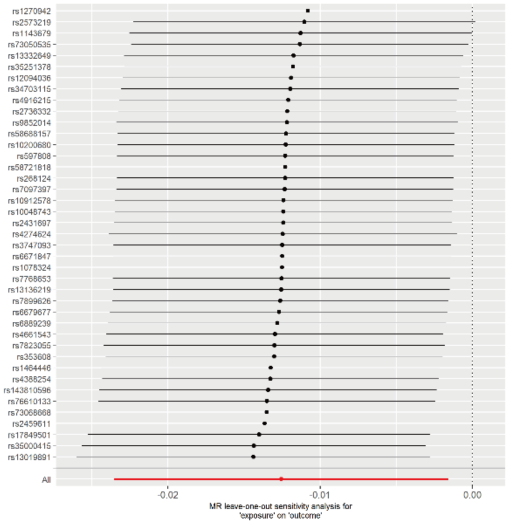

B-3

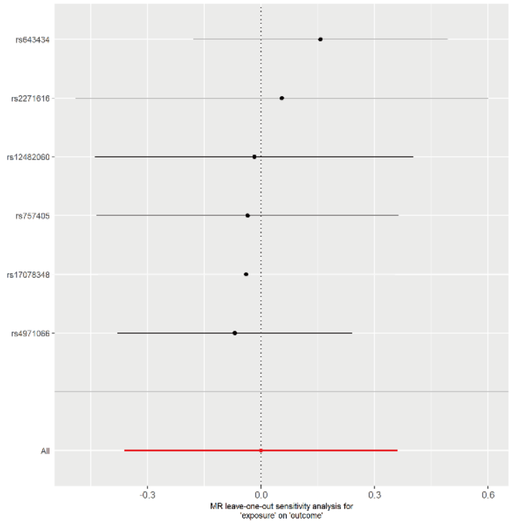

Figure S4. Leave-one-out plot of the causal relationships between SLE and COVID-19.

Legend: (A-1) MR estimates for SLE on severe COVID-19. (A-1) MR estimates for SLE on hospitalized COVID-19. (A-3) SLE on COVID-19. (B-1) MR estimates for severe COVID-19 on SLE. (B-2) Hospitalized COVID-19 on SLE. (B-3) COVID-19 on SLE.
