# Supplementary material for: No genetic causal association between systemic lupus erythematosus and COVID-19
Source: Front Immunol. 2023 May 18;14:1183570. doi: 10.3389/fimmu.2023.1183570 (PMC10232808; doi:10.3389/fimmu.2023.1183570)
Supplement: Supplementary file 2 [file Table_1.docx]

| **Supplementary Table 1. List of SLE Genetic instruments** | | | |  |  | |  | |  |  | |  | | |  |  |
| --- | --- | --- | --- | --- | --- | --- | --- | --- | --- | --- | --- | --- | --- | --- | --- | --- |
| **SNP** | **CHR** | **POS** | **effect_allele** | **other_allele** | **Beta** | **SE** | | **pval** | | | **EAF** | | **F value** |  |  |  |
| rs10048743 | 2 | 213890232 | G | T | 0.231 | 0.041 | | 2.04E-08 | | | 0.86 | | 31.741 |  |  |  |
| rs10200680 | 2 | 223961877 | T | C | -0.248 | 0.042 | | 4.96E-09 | | | 0.14 | | 34.863 |  |  |  |
| rs1078324 | 5 | 149202268 | A | C | -0.713 | 0.078 | | 7.11E-20 | | | 0.05 | | 83.551 |  |  |  |
| rs10912578 | 1 | 173251856 | A | G | 0.247 | 0.031 | | 1.65E-15 | | | 0.72 | | 63.479 |  |  |  |
| rs1143679 | 16 | 31276811 | A | G | 0.582 | 0.040 | | 5.03E-48 | | | 0.13 | | 211.684 |  |  |  |
| rs12094036 | 1 | 183558174 | C | T | -0.329 | 0.058 | | 1.37E-08 | | | 0.08 | | 32.174 |  |  |  |
| rs1270942 | 6 | 31918860 | G | A | 0.928 | 0.043 | | 1.45E-101 | | | 0.07 | | 465.716 |  |  |  |
| rs13019891 | 2 | 113829869 | T | G | -0.562 | 0.029 | | 1.65E-83 | | | 0.46 | | 375.525 |  |  |  |
| rs13136219 | 4 | 102743687 | T | C | -0.174 | 0.028 | | 3.50E-10 | | | 0.36 | | 38.614 |  |  |  |
| rs13332649 | 16 | 85966683 | G | A | -0.315 | 0.038 | | 5.43E-17 | | | 0.20 | | 68.709 |  |  |  |
| rs143123127 | 17 | 38007190 | A | G | 0.470 | 0.084 | | 2.23E-08 | | | 0.04 | | 31.304 |  |  |  |
| rs143810596 | 6 | 32390436 | G | T | -0.616 | 0.113 | | 4.41E-08 | | | 0.03 | | 29.714 |  |  |  |
| rs1464446 | 3 | 146601295 | T | G | -0.329 | 0.040 | | 2.79E-16 | | | 0.18 | | 67.645 |  |  |  |
| rs150180633 | 6 | 31010047 | T | C | 0.928 | 0.069 | | 2.66E-41 | | | 0.02 | | 180.867 |  |  |  |
| rs17849501 | 1 | 183542323 | T | C | 0.811 | 0.050 | | 1.81E-59 | | | 0.06 | | 263.066 |  |  |  |
| rs2431697 | 5 | 159879978 | C | T | -0.223 | 0.029 | | 2.60E-14 | | | 0.42 | | 59.126 |  |  |  |
| rs2459611 | 2 | 191939187 | C | T | -0.261 | 0.045 | | 7.62E-09 | | | 0.89 | | 33.637 |  |  |  |
| rs2573219 | 2 | 233288667 | C | A | 0.588 | 0.043 | | 1.13E-42 | | | 0.09 | | 186.974 |  |  |  |
| rs268124 | 2 | 65654364 | C | T | -0.186 | 0.032 | | 8.60E-09 | | | 0.72 | | 33.782 |  |  |  |
| rs2736332 | 8 | 11339965 | C | G | 0.278 | 0.032 | | 4.83E-18 | | | 0.26 | | 75.466 |  |  |  |
| rs34703115 | 2 | 40282854 | C | T | -0.616 | 0.105 | | 4.08E-09 | | | 0.04 | | 34.415 |  |  |  |
| rs35000415 | 7 | 128585616 | T | C | 0.588 | 0.042 | | 1.86E-45 | | | 0.10 | | 195.983 |  |  |  |
| rs35251378 | 19 | 10459969 | A | G | -0.236 | 0.032 | | 3.61E-13 | | | 0.28 | | 54.386 |  |  |  |
| rs353608 | 11 | 35101738 | A | G | -0.186 | 0.028 | | 2.93E-11 | | | 0.54 | | 44.124 |  |  |  |
| rs3747093 | 22 | 21984379 | A | G | 0.262 | 0.035 | | 2.88E-14 | | | 0.20 | | 56.031 |  |  |  |
| rs4274624 | 2 | 191958656 | C | T | 0.560 | 0.033 | | 9.73E-66 | | | 0.77 | | 287.946 |  |  |  |
| rs4388254 | 5 | 133428601 | T | C | 0.378 | 0.060 | | 3.71E-10 | | | 0.07 | | 39.687 |  |  |  |
| rs4661543 | 1 | 15229101 | T | G | -0.274 | 0.042 | | 9.40E-11 | | | 0.88 | | 42.556 |  |  |  |
| rs4916215 | 1 | 173314540 | C | T | -0.223 | 0.034 | | 5.07E-11 | | | 0.75 | | 43.014 |  |  |  |
| rs58688157 | 11 | 625085 | G | A | -0.223 | 0.034 | | 2.97E-11 | | | 0.26 | | 43.014 |  |  |  |
| rs58721818 | 6 | 138243739 | T | C | 0.658 | 0.076 | | 3.38E-18 | | | 0.02 | | 74.953 |  |  |  |
| rs597808 | 12 | 111973358 | G | A | -0.163 | 0.029 | | 3.51E-08 | | | 0.51 | | 31.589 |  |  |  |
| rs6671847 | 1 | 161478810 | A | G | 0.199 | 0.029 | | 6.64E-12 | | | 0.47 | | 47.084 |  |  |  |
| rs6679677 | 1 | 114303808 | A | C | 0.336 | 0.046 | | 4.55E-13 | | | 0.10 | | 53.349 |  |  |  |
| rs6889239 | 5 | 150457771 | C | T | 0.278 | 0.032 | | 2.19E-18 | | | 0.25 | | 75.466 |  |  |  |
| rs7097397 | 10 | 50025396 | A | G | -0.186 | 0.029 | | 8.60E-11 | | | 0.39 | | 41.133 |  |  |  |
| rs73050535 | 12 | 5012503 | T | C | -0.713 | 0.124 | | 9.11E-09 | | | 0.03 | | 33.060 |  |  |  |
| rs73068668 | 19 | 55763262 | A | G | -0.315 | 0.057 | | 4.40E-08 | | | 0.10 | | 30.538 |  |  |  |
| rs7768653 | 6 | 106574794 | C | T | 0.207 | 0.030 | | 3.11E-12 | | | 0.59 | | 47.606 |  |  |  |
| rs7823055 | 8 | 55511676 | G | T | 0.351 | 0.029 | | 1.64E-34 | | | 0.56 | | 146.481 |  |  |  |
| rs7899626 | 10 | 63825561 | T | C | 0.182 | 0.033 | | 4.19E-08 | | | 0.36 | | 30.414 |  |  |  |
| rs9274357 | 6 | 32632457 | T | C | 0.457 | 0.035 | | 1.28E-38 | | | 0.19 | | 170.474 |  |  |  |
| rs9852014 | 3 | 129084581 | G | A | 0.621 | 0.049 | | 2.26E-36 | | | 0.07 | | 160.603 |  |  |  |

Abbreviations: SNP: single nucleotide polymorphism; CHR: chromosome; EAF: effect allele frequency; SE: standard error of beta.

| **Supplementary Table 2. List of COVID-19 Genetic instruments** | | | | | |  | |  | |  | |  | |  | |  | |  |
| --- | --- | --- | --- | --- | --- | --- | --- | --- | --- | --- | --- | --- | --- | --- | --- | --- | --- | --- |
| **Exposure** | **SNP** | **CHR** | **POS** | **effect_allele** | **other_allele** | | **Beta** | | **SE** | | **pval** | | **EAF** | | **sample_N** | | **F value** | |
| Severe COVID-19 |  |  |  |  |  | |  | |  | |  | |  | |  | |  | |
|  | rs35081325 | 3 | 45889921 | A | T | | 0.626 | | 0.045 | | 5.75E-45 | | 0.075 | | 707407 | | 193.518 | |
|  | rs111837807 | 6 | 31121232 | T | C | | 0.295 | | 0.043 | | 5.66E-12 | | 0.100 | | 707407 | | 47.066 | |
|  | rs622568 | 7 | 54647894 | A | C | | 0.226 | | 0.037 | | 1.04E-09 | | 0.146 | | 468293 | | 37.309 | |
|  | rs2237698 | 7 | 107607902 | C | T | | 0.237 | | 0.040 | | 2.41E-09 | | 0.090 | | 707407 | | 35.106 | |
|  | rs10860891 | 12 | 103014757 | C | A | | -0.239 | | 0.040 | | 1.64E-09 | | 0.886 | | 468696 | | 35.700 | |
|  | rs2384074 | 12 | 113382977 | C | T | | 0.198 | | 0.028 | | 2.10E-12 | | 0.676 | | 707407 | | 50.005 | |
|  | rs77534576 | 17 | 47940666 | C | T | | 0.460 | | 0.075 | | 8.53E-10 | | 0.035 | | 707407 | | 37.618 | |
|  | rs2109069 | 19 | 4719443 | G | A | | 0.257 | | 0.028 | | 6.12E-20 | | 0.329 | | 707407 | | 84.246 | |
|  | rs13050728 | 21 | 34615210 | T | C | | -0.200 | | 0.029 | | 2.44E-12 | | 0.663 | | 707407 | | 47.562 | |
| Hospitalized COVID-19 |  |  |  |  |  | |  | |  | |  | |  | |  | |  | |
|  | rs35081325 | 3 | 45889921 | A | T | | 0.488 | | 0.032 | | 3.68E-54 | | 0.081 | | 1206629 | | 232.562 | |
|  | rs622568 | 7 | 54647894 | A | C | | 0.154 | | 0.026 | | 3.64E-09 | | 0.151 | | 496465 | | 35.083 | |
|  | rs505922 | 9 | 136149229 | T | C | | -0.112 | | 0.019 | | 4.42E-09 | | 0.650 | | 1206629 | | 34.748 | |
|  | rs2660 | 12 | 113357442 | G | A | | 0.116 | | 0.019 | | 2.01E-09 | | 0.690 | | 1206016 | | 37.274 | |
|  | rs2109069 | 19 | 4719443 | G | A | | 0.151 | | 0.020 | | 2.94E-14 | | 0.323 | | 1204013 | | 57.002 | |
|  | rs13050728 | 21 | 34615210 | T | C | | -0.168 | | 0.020 | | 7.44E-17 | | 0.653 | | 1203392 | | 70.560 | |
| COVID-19 infection |  |  |  |  |  | |  | |  | |  | |  | |  | |  | |
|  | rs4971066 | 1 | 155105882 | T | G | | -0.077 | | 0.013 | | 1.02E-08 | | 0.178 | | 1501994 | | 35.083 | |
|  | rs2271616 | 3 | 45838013 | G | T | | 0.156 | | 0.015 | | 3.61E-25 | | 0.118 | | 1484622 | | 108.160 | |
|  | rs17078348 | 3 | 45847241 | A | G | | 0.092 | | 0.016 | | 1.20E-08 | | 0.100 | | 1507972 | | 33.062 | |
|  | rs10936744 | 3 | 101433440 | C | T | | -0.063 | | 0.010 | | 3.51E-10 | | 0.359 | | 1588784 | | 39.690 | |
|  | rs643434 | 9 | 136142355 | A | G | | -0.101 | | 0.010 | | 1.29E-23 | | 0.629 | | 1554714 | | 102.010 | |
|  | rs757405 | 12 | 113406945 | T | A | | 0.069 | | 0.011 | | 1.64E-10 | | 0.709 | | 1312020 | | 39.347 | |
|  | rs12482060 | 21 | 34611571 | C | G | | 0.062 | | 0.011 | | 3.958E-09 | | 0.338 | | 1313591 | | 31.769 | |

Abbreviations: SNP: single nucleotide polymorphism; CHR: chromosome; EAF: effect allele frequency; SE: standard error of beta.

| **Supplementary Table 3. MR estimate results of SLE on COVID-19** | | | | | | | |  | |  | |  | |  | |  | |  | | |  | |  |  | |  |
| --- | --- | --- | --- | --- | --- | --- | --- | --- | --- | --- | --- | --- | --- | --- | --- | --- | --- | --- | --- | --- | --- | --- | --- | --- | --- | --- |
| **Outcome** | **Methods** | **Nsnp** | **beta** | **SE** | ***P* value** | **OR** | **or_lci95** | | **or_uci95** | | **Horizontal pleiotropy** | | | | | | | | | **Heterogeneity** | | | | |  |  |
|  |  |  |  |  |  |  |  |  |  |  | **MR-Egger regression** | | | | | | **MR-PRESSO** | | | **Cochran’s *Q*** | | ***P* value** | | |  |  |
|  |  |  |  |  |  |  |  |  |  |  | **Egger intercept** | | **SE** | | ***P* value** | | **Global test *P* value** | | **Pleiotropic SNP** |  |  |  |  |  |  |  |
| Severe COVID-19 | Weighted median | 41 | -0.026 | 0.025 | 0.308 | 0.975 | 0.928 | | 1.024 | | -0.001 | | 0.014 | | 0.940 | | 0.146 | | no outlier | 50.070 | | 0.132 | | |  |  |
|  | MR Egger | 41 | -0.036 | 0.040 | 0.368 | 0.964 | 0.892 | | 1.043 | |  | |  | |  | |  | |  |  | |  | | |  |  |
|  | Weighted mode | 41 | -0.021 | 0.030 | 0.476 | 0.979 | 0.924 | | 1.037 | |  | |  | |  | |  | |  |  | |  | | |  |  |
|  | IVW | 41 | -0.039 | 0.019 | 0.040 | 0.962 | 0.927 | | 0.998 | |  | |  | |  | |  | |  |  | |  | | |  |  |
| Hospitalized COVID-19 | Weighted median | 41 | -0.015 | 0.015 | 0.338 | 0.985 | 0.956 | | 1.016 | | -0.008 | | 0.009 | | 0.371 | | 0.339 | | no outlier | 43.669 | | 0.318 | | |  |  |
|  | MR Egger | 41 | 0.003 | 0.024 | 0.918 | 1.003 | 0.956 | | 1.052 | |  | |  | |  | |  | |  |  | |  | | |  |  |
|  | Weighted mode | 41 | -0.011 | 0.021 | 0.595 | 0.989 | 0.949 | | 1.030 | |  | |  | |  | |  | |  |  | |  | | |  |  |
|  | IVW | 41 | -0.017 | 0.012 | 0.148 | 0.983 | 0.961 | | 1.006 | |  | |  | |  | |  | |  |  | |  | | |  |  |
| COVID-19 infection | Weighted median | 41 | -0.015 | 0.008 | 0.066 | 0.985 | 0.969 | | 1.001 | | -0.001 | | 0.004 | | 0.746 | | 0.798 | | no outlier | 32.885 | | 0.780 | | |  |  |
|  | MR Egger | 41 | -0.009 | 0.012 | 0.440 | 0.991 | 0.968 | | 1.014 | |  | |  | |  | |  | |  |  | |  | | |  |  |
|  | Weighted mode | 41 | -0.020 | 0.012 | 0.102 | 0.980 | 0.957 | | 1.004 | |  | |  | |  | |  | |  |  | |  | | |  |  |
|  | IVW | 41 | -0.013 | 0.006 | 0.025 | 0.988 | 0.977 | | 0.998 | |  | |  | |  | |  | |  |  | |  | | |  |  |

Abbreviations: SNP: single nucleotide polymorphism; CHR: chromosome; EAF: effect allele frequency; SE: standard error of beta; IVW: Inverse variance weighted.

| **Supplementary Table 4. MR estimate results of COVID-19 on SLE** | | | | | | | |  | |  | |  | |  | |  | |  | |  | |  | |  | |  |
| --- | --- | --- | --- | --- | --- | --- | --- | --- | --- | --- | --- | --- | --- | --- | --- | --- | --- | --- | --- | --- | --- | --- | --- | --- | --- | --- |
| **Exposure** | **Methods** | **Nsnp** | **Beta** | **SE** | ***P* value** | **OR** | **or_lci95** | | **or_uci95** | | **Horizontal pleiotropy** | | | | | | | | | | **Heterogeneity** | | | |  |  |
|  |  |  |  |  |  |  |  |  |  |  | **MR-Egger regression** | | | | | | **MR-PRESSO** | | | | **Cochran’s *Q*** | | ***P* value** | |  |  |
|  |  |  |  |  |  |  |  |  |  |  | **Egger intercept** | | **SE** | | ***P* value** | | **Global test *P* value** | | **Pleiotropic SNP** | |  |  |  |  |  |  |
| Severe COVID-19 | Weighted median | 7 | -0.039 | 0.070 | 0.577 | 0.962 | 0.839 | | 1.103 | | 0.075 | | 0.082 | | 0.430 | | 0.423 | | no outlier | | 4.129 | | 0.389 | |  |  |
|  | MR Egger | 7 | 0.014 | 0.258 | 0.958 | 1.014 | 0.612 | | 1.680 | |  | |  | |  | |  | |  | |  | |  | |  |  |
|  | Weighted mode | 7 | -0.057 | 0.075 | 0.473 | 0.944 | 0.816 | | 1.093 | |  | |  | |  | |  | |  | |  | |  | |  |  |
|  | IVW | 7 | 0.044 | 0.101 | 0.664 | 1.045 | 0.858 | | 1.273 | |  | |  | |  | |  | |  | |  | |  | |  |  |
| Hospitalized COVID-19 | Weighted median | 5 | -0.104 | 0.096 | 0.278 | 0.901 | 0.746 | | 1.088 | | 0.042 | | 0.030 | | 0.256 | | 0.553 | | no outlier | | 2.747 | | 0.601 | |  |  |
|  | MR Egger | 5 | 0.035 | 0.150 | 0.832 | 1.035 | 0.772 | | 1.388 | |  | |  | |  | |  | |  | |  | |  | |  |  |
|  | Weighted mode | 5 | -0.074 | 0.112 | 0.544 | 0.929 | 0.745 | | 1.157 | |  | |  | |  | |  | |  | |  | |  | |  |  |
|  | IVW | 5 | -0.137 | 0.086 | 0.109 | 0.872 | 0.737 | | 1.031 | |  | |  | |  | |  | |  | |  | |  | |  |  |
| COVID-19 infection | Weighted median | 7 | -0.066 | 0.187 | 0.723 | 0.936 | 0.648 | | 1.351 | | -0.015 | | 0.071 | | 0.846 | | 0.029 | | rs10936744 | | 18.359 | | 0.005 | |  |  |
|  | MR Egger | 7 | 0.020 | 0.757 | 0.980 | 1.020 | 0.231 | | 4.500 | |  | |  | |  | |  | |  | |  | |  | |  |  |
|  | Weighted mode | 7 | -0.081 | 0.198 | 0.698 | 0.923 | 0.626 | | 1.360 | |  | |  | |  | |  | |  | |  | |  | |  |  |
|  | IVW | 7 | -0.125 | 0.241 | 0.605 | 0.883 | 0.551 | | 1.415 | |  | |  | |  | |  | |  | |  | |  | |  |  |
| COVID-19 infection with no MR-PRESSO outliers | Weighted median | 6 | -0.040 | 0.183 | 0.826 | 0.961 | 0.670 | | 1.376 | | 0.051 | | 0.055 | | 0.401 | | 0.200 | | no outlier | | 8.264 | | 0.142 | |  |  |
|  | MR Egger | 6 | -0.488 | 0.552 | 0.427 | 0.614 | 0.208 | | 1.811 | |  | |  | |  | |  | |  | |  | |  | |  |  |
|  | Weighted mode | 6 | -0.070 | 0.205 | 0.747 | 0.932 | 0.624 | | 1.394 | |  | |  | |  | |  | |  | |  | |  | |  |  |
|  | IVW | 6 | -0.001 | 0.184 | 0.998 | 0.999 | 0.697 | | 1.434 | |  | |  | |  | |  | |  | |  | |  | |  |  |

Abbreviations: SNP: single nucleotide polymorphism; CHR: chromosome; EAF: effect allele frequency; SE: standard error of beta; IVW: Inverse variance weighted.
